# MRI-guided stereotactic body radiation therapy for very elderly patients with primary lung cancer: a retrospective analysis of safety and efficacy profiles

**DOI:** 10.1007/s11547-025-02056-1

**Published:** 2025-07-22

**Authors:** Luca Boldrini, Angela Romano, Ilaria Castanò, Antonella Martino, Filippo Lococo, Giuseppe Cicchetti, Matteo Nardini, Giulia Panza, Lorenzo Placidi, Giuditta Chiloiro

**Affiliations:** 1 Dipartimento di Diagnostica per Immagini e Radioterapia Oncologica, Fondazione Policlinico Universitario “A. Gemelli” IRCCS, Largo Agostino Gemelli 8, 00168 Rome, Italy; 2 Università Cattolica del Sacro Cuore, Largo Francesco Vito 1, 00168 Rome, Italy; 3 Unità di Chirurgia Toracica, Fondazione Policlinico Universitario “A. Gemelli” IRCCS, Largo Agostino Gemelli 8, 00168 Rome, Italy

**Keywords:** Lung cancer, Stereotactic body radiation therapy, Magnetic resonance-guided radiation therapy, Elderly patients

## Abstract

**Purpose:**

Non-small cell lung cancer is the most common malignancy of the lung, with over 40% of the cases in patients aged 75 years or older. Many of these patients are inoperable due to comorbidities, limiting treatment options. Stereotactic body radiotherapy (SBRT) offers a curative alternative, achieving local control (LC) rates similar to surgery with manageable toxicity. This retrospective analysis aimed to investigate the efficacy and safety of MRI-guided SBRT (MRIgSBRT) for elderly lung cancer patients.

**Materials and methods:**

Data of patients aged ≥ 75 years, treated in our Institution between 2017 and 2023, were retrospectively collected. Survival curves for local recurrence-free survival (LRFS), progression-free survival and overall survival (OS) were estimated using the Kaplan–Meier method. Toxicity was assessed using the Common Terminology Criteria for Adverse Events (CTCAE version 5.0) scale.

**Results:**

The study included 38 patients with a total of 45 lung lesions, median age of 82 years (range 75–87). The median total radiotherapy dose was 62,5 Gy (range 24–75 Gy) delivered in 5 fractions (range 3–8). The median follow-up was of 16.9 months (range 0,97–66,7). The 1-, 2- and 3-year OS rates were 98% 96% and 80%, respectively, while the 1-, 2- and 3-year LRFS was 97,5%. Six patients (15.78%) and one patient (2.63%) had late G1 radiation-induced pneumonia and G2 dyspnoea, respectively.

**Conclusions:**

MRIgSBRT is a valid therapeutic option for patients aged ≥ 75 with comorbidities, frailty and risk factors limiting their performance status and eligibility for invasive treatments, offering good LC and favourable toxicity profile.

## Introduction

Lung cancer is the leading cause of cancer-related mortality in the general population accounting for about 22% of all cancer deaths [1, 2]. The most common subtype is non-small cell lung cancer (NSCLC) [3]. Although in the general population NSCLC diagnosis occurs at a later metastatic stage [4], a cross-sectional epidemiological analysis [5] suggested a slight decrease of incidence of stage IV (from 21.7 to 19.6 per 100 000) and an opposite rise in both prevalence and incidence of stage I particularly among patients aged 65 years or older, compatible with an improved evaluation of incidental nodules. In this latter study, prior to the approval of immunotherapy as an alternative or additional therapeutic option, almost half received a single modality treatment (i.e. surgery, radiation, chemotherapy) and 27.7% of the patients received multiple treatments, with the remaining 23% of the population receiving no therapy (stage IV with 77.5% aged > 65 years old) [5].

Generally, the proportions of patients with stage I to IIIA receiving surgery alone as initial option decreased, whereas those with stage I–II diseases receiving radiotherapy alone have been characterized by an overall increase, more pronounced in elderly patients (17.4–30.2%) [5]. This approach results to be particularly preferred in very elderly patients (aged 75 years or older), whose multimorbidity and higher frailty can hinder the standard surgical treatment of the lesion, despite the earlier disease stage [6]. In fact, in accordance with the latest ESMO guidelines [41], pretreatment risk assessment should be established; medical operability should be evaluated on the basis of comorbidities and cardiopulmonary fitness, measuring the recalibrated thoracic revised cardiac risk index (RCRI) and the predicted post-operative FEV_1_ and DLCO.

Thanks to its characteristics, Stereotactic body radiation therapy (SBRT) has emerged as a potential therapeutic alternative in unfit older individuals with early-stage NSCLC, delivering high doses of radiation in few fractions (usually 3 to 8) with an additional daily image-guided re-localization generally based on highly precise robotic solutions [7].

Several studies [7–11] have underlined the safety and efficacy profiles of SBRT in disease control in very elderly patients with acceptable toxicity rates, encompassing likewise individuals with centrally located tumours [12]. Furthermore, the introduction of magnetic resonance-guided radiation therapy (MRIgRT) allowed an improved soft tissue visualization with real-time target therapy volumes gating and daily adaptation to minimize toxicities and optimize target coverage [13–17].

Purpose of this retrospective analysis is to assess the clinical effectiveness of MRIgSBRT in patients aged 75 years or older affected by NSCLC and treated in our Institution with both curative and palliative intent. Data related to local recurrence-free survival (LRFS), progression-free survival (PFS) and overall survival (OS) were collected, as well as the associated toxicity profile.

## Materials and methods

### Patients

We retrospectively analysed and collected the data of patients aged 75 years or older at the starting date of SBRT treatment for a diagnosis of NSCLC and SCLC in a minority of cases, in our centre between 2017 and 2023. The individuals were identified from the electronic database of our Institution.

The lesions included in this study were both early-stage tumours (cT1-2 N0 M0) and advanced neoplasms (cT3-4 N0), according to the Union for International Cancer Control (UICC) TNM classification 8th edition [18]. The study also included patients with induced oligometastatic disease (stage IVA e IVB) [19] and others who presented lung disease recurrence after undergoing surgery. In particular, stage IVA cases were treated in all disease sites, while stage IVB patients were managed in an oligoprogressive setting.

The staging of these patients also included the pathological characterization of disease, when feasible. Lesions which were not biopsied, due to comorbidities and frail conditions of the referred patients, were also included. Disease staging was further completed by a contrast-enhanced total body computed tomography (CT) and positron emission tomography (PET-CT).

Furthermore, the pretreatment Charlson Comorbidity Index [20] was calculated by processing data extracted from the hospital database (i.e. age, sex, median follow-up), including the cancer score, stratified for localized or metastatic solid tumour. All patients were evaluated as inoperable by our multidisciplinary tumour board after examining respiratory function comorbidities, disease burden or general performance status.

### Treatment

At the start of treatment evaluation, all patients completed a MRI compatibility questionnaire and were evaluated for the presence of non-MRI compatible devices (e.g. pacemakers), severe claustrophobia, cognitive impairment, ability to maintain a stable position for 20–25 min and respiratory control. Patients with contraindications or significant issues during simulation (e.g. severe claustrophobia or refusal) were excluded from MRIgSBRT.

All patients were immobilized in a supine position using a customized repositioning device Fluxboard (Fluxboard™, MacroMedics, the Netherlands) and underwent magnetic resonance imaging (MRI) simulation on a 0.35 T hybrid MRI-guided unit (MRIdian, View. Ray Technologies, USA).

Initially, the Cobalt-60 configuration was utilized, featuring a tri-cobalt-60 gantry integrated with on-board MRI imaging [21]. Following an upgrade in February 2018, the system was transitioned to the Linac version combining a 0.35 T MRI scanner with a 6-MV flattening filter-free (FFF) linear accelerator (Linac) [22]. All plans were performed with the MRIdian treatment planning system using a Monte Carlo calculation algorithm with a step-and-shoot intensity modulated radiation technique (IMRT) [23], considering the influence of 0.35 T on the dose calculation. True fast imaging (TRUFI) MR scans were obtained with different acquisition protocols either free breathing (FB) or inspiration breath-hold (BHI) to assess tumour and organs at risk (OARs) motion.

A real-time cine TRUFI MRI sequences (4/8 frames/s) was used to assess reproducibility and patient tolerance to gating during the simulation phases. Several delivery related parameters were defined during the simulation, such as the gating target structure (which could be the lesion itself or a surrogate), the boundary values tracking algorithms confidence rating and percentage of region of interest (ROI%) outside the boundary.

A concomitant standard simulation with a standard non-contrast-enhanced simulation computed tomography (CT) was performed to obtain the electron density information for the dose calculation. The deformable co-registration of the CT on the primary imaging simulation allowed the contouring of the lesion and the adjacent structures according to the RTOG guidelines [24, 25], using the informative features obtained from the additional diagnostic imaging tools (diagnostic CE CT or ^18^FDG PET-CT) for the delination of the gross tumour volume (GTV). The clinical target volume (CTV) was considered equal to the GTV, while a 3 to 5 mm margin was applied to generate the planning target volume (PTV), depending on clinical judgement and observed intrafraction motion.

Dose PTV coverage was assessed according to the International Commission on Radiation Units and Measurements (ICRU) [26, 27] recommendations and constraints suggested by the American Association of Physicists in Medicine (AAPM) Task Group 101 [28] were used for dose evaluation to OARs.

The indication for Stereotactic MRI-guided Online Adaptive Therapy (SMART) was given by the attending physician and treatment plan re-optimization with deformable contour registration, and adjustment dose prediction and secondary Monte Carlo-based quality assurance (QA) were performed accordingly when deemed necessary [29, 30]. After having selected the most appropriate plane for gating and having specified the recalled parameters set in the simulation phase, the treatment was delivered with direct gating and online 4–8 frames per second cine-MRI monitoring. The conformity index (CI) and the homogeneity index (HI) of the target lesions were measured according to the RTOG definitions [31].

#### Follow-up and toxicity evaluation

Clinical and toxicity data were retrospectively collected. Local and systemic response to treatment were assessed in line with the RECIST criteria according to a follow-up schedule that included instrumental re-evaluation every 3–6 months contrast-enhanced CT or ^18^FDG PET-CT, depending on clinical judgement [32]. Toxicities were considered acute if recorded during or up to 90 days after the end of treatment or late if afterwards and recorded using the Common Terminology Criteria for Adverse Events (CTCAE scale version 5.0)[33].

### Statistical analysis

We evaluated the Kaplan–Meier survival curves investigating overall survival (OS) local recurrence-free survival (LRFS) and progression-free survival (PFS). We defined the OS variable as the time interval between the start date of the treatment and the last follow-up date for each patient. The LRFS was determined as the period ranging from the end of therapy to the reported date of local recurrence, whereas the PFS was identified as the temporal length between the end date of the treatment and the time of progression of disease distant from the primarily treated site.

This is a retrospective, observational study for which ethical approval from the Fondazione Gemelli Ethics Committee is not required.

## Results

A total of 38 patients and 45 lesions were treated with MRIgSBRT in our institution between June 2017 and August 2023. Within this sample, very elderly (75 years old or more) individuals have been identified with a median age of 82 years (range 75–87).

We included comparable groups of female (13, 34.21%) and male (25, 65.79%) patients with pathologically proven NSCLC (17, 44.73% adenocarcinoma; 2, 5.26% squamous cell carcinoma; 1, 2.63% mixed adenocarcinoma), unclassified NSCLC (2, 5.26%), LC-NEC (1, 2.63%), SCLC (2, 5.26%) and N/A (15, 39.47%). The Charlson score [20] showed a median of 7 (range 5–12). The median prescribed dose was 62.5 Gy (range 24–75 Gy) in 5 fractions (3–8).

In total, 17 adaptive fractions (7.42% on a total number of 229 fractions) were delivered in 9 (23.68%) patients. The compliance of the treated patients was indirectly estimated upon evaluation of the fractions interrupted for each delivery plan showing a median value of 1 suspension every 5 fractions (1/5) with the range of 1/8—3/5 for 17 (37.8%) irradiated lesions, mainly due to temporary muscular fatigue. Patients, tumour and treatment characteristics are summarized in Table 1.

**Table 1:** Baseline patient and tumour characteristics

Characteristics	Number (%)
Patients (*n*)	38
Lesions (*n*)	45
*MRIdian*	
Tri-cobalt-60	6 (15.79)
Linac	32 (84.21)
Age (years), median (range)	82 (75–87)
*Gender*	
Female/male	13/25
Follow-up (months), median (range)	16.87 (0.97–66.70)
Charlson score, median (range)	7 (5–12)
5	4 (10.52)
6	13 (34.21)
7	7 (18.42)
8	5 (13.16)
9	1 (2.63)
10	6 (15.79)
11	1 (2.63)
12	1 (2.63)
*Stage grouping (TNM 8th)*	
IA	17 (44.73)
IB	6 (15.78)
IIB	2 (5.26)
IIIA	3 (7.89)
IVA	3 (7.89)
IVB	2 (5.26)
N/A	1 (2.63)
Relapse after surgery	3 (7.89)
Induced oligometastatic disease	1 (2.63)
Lesion maximum diameter (mm), median (range)	15 (6–40)
GTV (CC), median (range)	3.6 (0.4–61)
PTV (CC), median (range)	10.05 (1.9–83.9)
*Histology (n)(%)*	
Adenocarcinoma	17 (44.73)
SCLC	2 (5.26)
Mixed adenocarcinoma	1 (2.63)
Unclassified NSCLC	2 (5.26)
LC-NEC	1 (2.63)
N/A	15 (39.47)
Prescribed dose (Gy), median (range)	62.5 (24–75)
24 in 3 fractions	1 (2.22)
50 in 5 fractions	36 (80)
55 in 5 fractions	6 (13.33)
60 in 8 fractions	2 (4.44)
*Normalization*	
Dmean	13 (28.9)
80% isodose	32 (71.1)
BED, median (range)	117.19 (36–136.07)
*Late Toxicity max (CTCAEv5)*	
Grade 1	6 (15.78)
Grade 2	1 (2.63)

*GTV* gross tumour volume, *PTV* planning target volume, *NSCLC* non-small cell lung cancer, *LC-NEC* large cell neuroendocrine cancer, *SCLC* small cell lung cancer, *N/A* not applicable, *BED* biologically effective dose

**Fig. 1:**
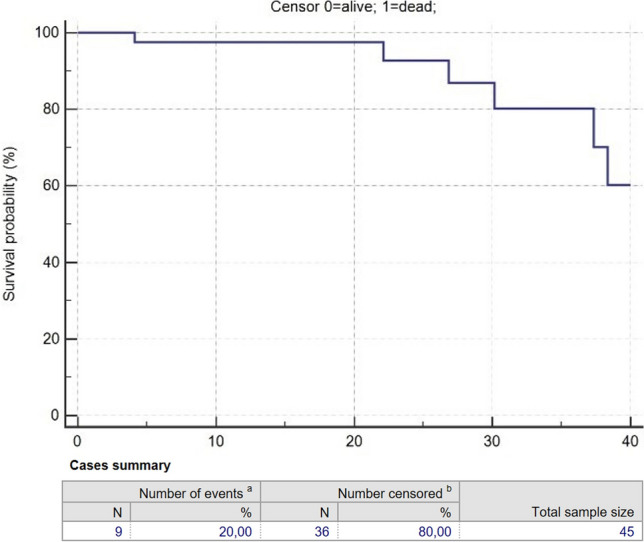
Overall survival (OS) rates over 40 months after MRIgSBRT

**Fig. 2:**
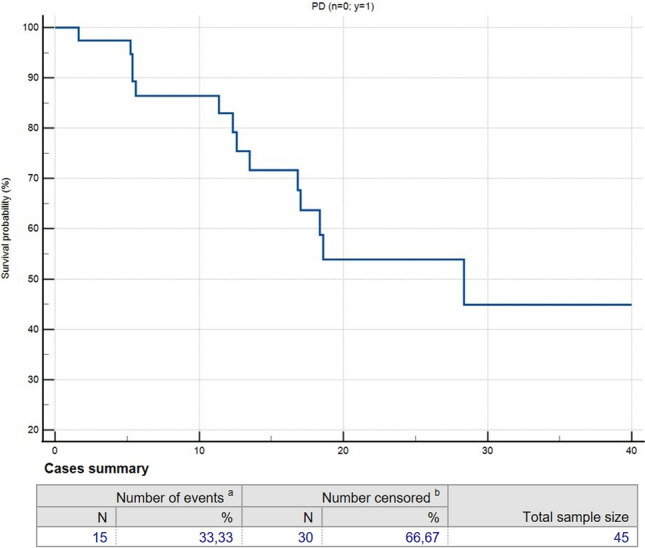
Progression-free survival (PFS) rates over 40 months after MRIgSBRT

**Fig. 3:**
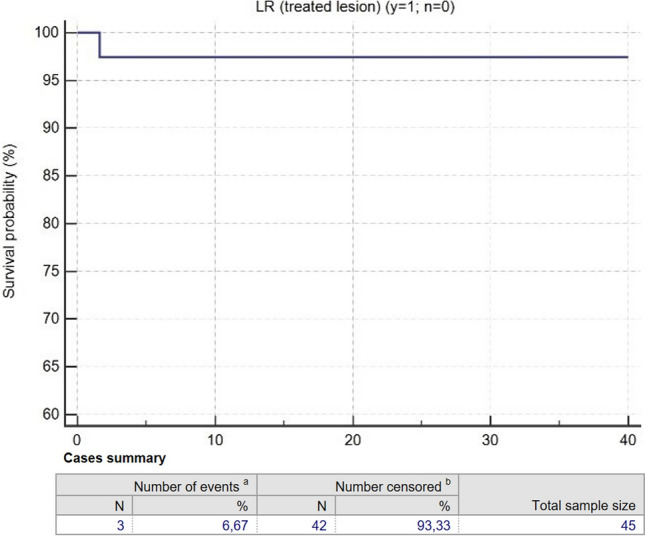
Local recurrence-free survival (LRFS) rates over 40 months after MRIgSBRT

The median follow-up period after SBRT was 16.87 months (0.97–66.70). No acute toxicities have been reported, while late toxicities were G1 post-actinic asymptomatic lung consolidations in six (15.78%) patients and G2 dyspnoea in one (2.63%) patient (Table 1). The analysed data demonstrated 1-, 2- and 3-year OS rates of 98%, 96% and 80% (Fig. 1), respectively, with a progression of disease (PD) established by 1-, 2- and 3-year PFS rates of 82%, 51% and 45% (Fig. 2), respectively, and the local recurrence (LR) evaluated through 1-, 2- and 3-year LRFS of 97.5% (Fig. 3). Univariate analysis did not show any significant predictor of LR and OS. Survival rates are reported in Table 2.

**Table 2:** Survival rates after MRIgSBRT

Variable	1 year	2 years	3 years
Overall survival (%)	98	96	80
Progression-free survival (%)	82	51	45
Local recurrence-free survival	97.5	97.5	97.5

## Discussion

This study was the first analysis upon the use of SBRT with real-time MRI guidance for the treatment of primary lung cancer in very elderly patients. It showed that this approach can ensure acceptable survival rates with good tolerability in a population with multiple comorbidities and limited therapeutic offering.

Current guidelines [34] standardize the treatment for early stages NSCLC with a first-line surgical resection. In this context, elderly patients with proven lung cancer are not denied pulmonary resection on the basis of age alone but are often judged inoperable owing to comorbidities and other frail conditions. When considered medically operable, surgical approach is associated with a higher risk of morbid complications and mortality both intra- and post-operatively [6]. This risk further rises with increased comorbidity scores [35]. More particularly, Mellemgaard and colleagues reported that Charlson score was > 2 in only 8% of patients aged < 60 years of their cohort and reached 26% and 43% in patients aged 60–69 and 70–79 years, respectively, in line with our experience [36].

Therefore, given the fact that comorbidities increase and associated surgical survival decreases with age, the population of very elderly patients (75 years old or more) greatly benefit from the introduction of advanced SBRT as therapeutic alternative.

Several studies on elderly patients have shown that SBRT can achieve a good and safe therapeutic response: for instance, Watanabe et al. [11] reported the outcomes of 162 elderly patients aged ≥ 80 years with pathologically proven early-stage non-small cell lung cancer demonstrating 1-, 3- and 5-year OS of 88.9, 68.3 and 47.5%; PFS rates of 81.0, 58.9 and 38.2%; and LC curves of 98.4, 90.1 and 87.4%, respectively.

Shu et al. [10] examined the use of SBRT on 68 elderly patients (≥ 75 years) with early-stage non-small cell lung cancer showing 1-, 3- and 5-year OS rates of 92.6, 77.2 and 59.1%; and 1-year, 3-year and 5-year LC rates of 95.6, 88.9 and 85.6%, respectively.

Other studies [37–39], despite a high Charlson score of the investigated elderly patients, underlined a good 3-year OS ranging from 40.7 to 53% and an adequate LC between 83 and 100%. In this study, the results of MRIgSBRT approach were promising and comparable with the reports mentioned above considering 1-, 2- and 3-year OS of 98, 96 and 80% PFS of 82, 51, 45% and LRFS of 97.5%, respectively. The tolerability profile of SBRT in this study is acceptable with 10.26% of patients (4) with 15.78% of patients (6) with G1 and 2.63% (1) G2 late toxicities. The complications we observed were even more limited, when compared to the findings reported in the review published by Nguyen et al. [35] in which acute G3 pneumonitis was in elderly patients in the range of 2.1–10%.

The severity of these adverse effects recorded within 90 days from the treatment was higher also in the group of patients analysed by Watanabe et al. [9], reporting G3 radiation pneumonitis (RP) in three patients and grade 5 RP in one individual. The increased efficacy and safety profiles determined in this study can probably be related to the use of the MRIgSBRT delivery technology, whose feasibility has been already defined by our group for elderly cancer individuals [40]; howbeit, further studies are needed to evaluate these findings, overcoming the intrinsic quantitative limitations of our samples.

This advanced technology has been proved to have also an efficient role in other population groups with central and ultracentral lung lesions [17] with 1-year LC, OS and PFS of 87, 82 and 54%, respectively, and a higher safety profile with 4.3% of late G3 toxicities if compared to some studies with CT based SBRT reporting tardive grade 3 to 5 toxicities as high as 30% [42, 43].

Despite faster due to the last technological developments, the MRIgSBRT delivery process still requires a higher compliance by the patient compared to standard radiation techniques, including MRI compatibility (e.g. presence of non-MRI compatible devices, claustrophobia, major cognitive impairment), breathing control and patient’s permanence in a stable position for an extended time that can reach 20–25 min per fraction.

This study presents some limitations, determined by the intrinsic drawbacks of a retrospective mono-centric analysis of a small sample size. Moreover, the results pointed out in the survival curves should be interpreted with caution, considering the heterogeneity of examined lung cancer lesions, which also included some stage III and IV patients. Nevertheless, this cohort represents a good picture of the feasibility and tolerability profile of MRIgSBRT treatment for lung malignancies if compared with other available techniques for patients’ cohort in a similar setting: Franceschini et al. [44], indeed, reported several cases of acute and late toxicities after volumetric modulated arc therapy (VMAT) in elderly patients with inoperable stage III NSCLC, specifically 61% of acute G1-2 and 32% of tardive G1-2 events. These initial findings may suggest a favourable therapeutic option for selected patients’ groups.

## Conclusions

This study aimed at examining the therapeutic contribution of MRIgSBRT in very elderly patients with no other valid treatment options. The resulting findings suggest the positive feasibility and toxicity profile of this approach for the management of complex patients aged 75 years or more, a niche population who often risks being undertreated and addressed to palliative care only.

## Data Availability

The datasets analysed during the current study are available from the corresponding author on reasonable request.
